# Targeting lipid metabolic reprogramming to alleviate diabetic kidney disease: molecular insights and therapeutic strategies

**DOI:** 10.3389/fimmu.2025.1549484

**Published:** 2025-04-25

**Authors:** Wei Yu, Yang Haoyu, Zhou Ling, Hang Xing, Xie Pengfei, Wang Anzhu, Zhang Lili, Zhao Linhua

**Affiliations:** ^1^ Institute of Metabolic Diseases, Guang’anmen Hospital, China Academy of Chinese Medical Sciences, Beijing, China; ^2^ Graduate College, Beijing University of Chinese Medicine, Beijing, China; ^3^ Chinese-Japanese Friendship Hospital, Beijing, China; ^4^ Department of Endocrinology, The Affiliated Hospital of Changchun University of Chinese Medicine, Changchun, Jilin, China

**Keywords:** diabetic kidney disease, lipid metabolic reprogramming, hypoxia-inducible factor-1α, inflammation, lipotoxicity

## Abstract

Diabetic kidney disease (DKD) is one of the major complications of diabetes, and its pathological progression is closely associated with lipid metabolic reprogramming. Under diabetic conditions, renal cells undergo significant lipid metabolic abnormalities, including increased lipid uptake, impaired fatty acid oxidation, disrupted cholesterol efflux, and enhanced lipid catabolism, as adaptive responses to metabolic stress. These changes result in the accumulation of lipids such as free fatty acids, diacylglycerol, and ceramides, leading to lipotoxicity that triggers inflammation and fibrosis. Hypoxia in the DKD microenvironment suppresses fatty acid oxidation and promotes lipid synthesis through the HIF-1α pathway, while chronic inflammation exacerbates lipid metabolic disturbances via inflammatory cytokines, inflammasomes, and macrophage polarization. Targeting lipid metabolism represents a promising therapeutic strategy for alleviating DKD; however, further clinical translational studies are warranted to validate the efficacy and safety of these approaches.

## Graphical Abstract

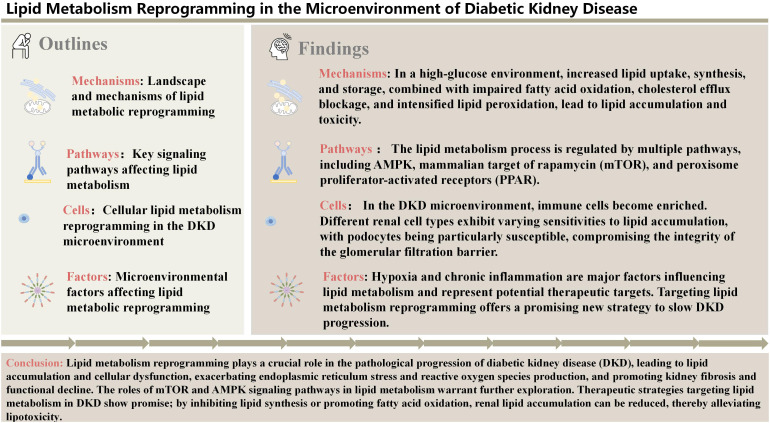

## 1 Introduction

Diabetic kidney disease (DKD) is one of the most common microvascular complications of diabetes, with approximately 40% of diabetic patients eventually developing DKD. It has become a leading cause of end-stage renal disease (ESRD) worldwide (1). As the prevalence of diabetes continues to rise, the incidence of DKD is also increasing, placing a significant burden on global public health systems. Although substantial progress has been made in understanding the pathological mechanisms of DKD, including aspects such as glucose metabolism, oxidative stress, and inflammation, its complex pathophysiology remains incompletely understood. In particular, the critical role of metabolic abnormalities in the onset and progression of DKD warrants further investigation (2, 3). In recent years, increasing evidence has highlighted the close association between metabolic dysregulation, particularly lipid metabolism abnormalities, and the pathological progression of DKD (4).

Significant changes in lipid metabolism in the context of diabetes have been widely reported. Specifically, insulin resistance and hyperglycemia in diabetic patients are often associated with increased free fatty acids, impaired adipose tissue function, and disrupted fatty acid oxidation, leading to lipid accumulation in tissues such as the liver, muscles, and kidneys (5, 6). Lipotoxicity is particularly pronounced in renal tissue, with numerous studies confirming that fatty acids and their metabolites, such as diacylglycerol (DAG) and ceramide, interfere with cellular function through various pathways, promoting inflammation and fibrosis, thereby accelerating the progression of DKD (7).

The kidney is a complex organ, and maintaining the stability of its microenvironment requires interactions among various cell types, including processes such as signal transduction and material exchange (8). The renal microenvironment consists of several cell types, including tubular epithelial cells, glomerular endothelial cells, podocytes, mesangial cells, and local immune cells (e.g., macrophages) (9). In the diabetic state, the metabolism and function of these cells undergo significant changes, with particular attention being drawn to the impact of lipid metabolism reprogramming on the renal microenvironment. Studies have shown that renal tubular epithelial cells are the most metabolically active cell type in the kidney, relying on fatty acid oxidation for energy. When diabetes induces lipid metabolic dysregulation, energy supply to renal tubular epithelial cells is limited, leading to metabolic reprogramming and accumulation of lipids and lipotoxicity, which in turn causes structural and functional changes in the kidney (10). Furthermore, inflammation and hypoxia within the renal microenvironment significantly contribute to the abnormal regulation of lipid metabolic pathways, and these factors collectively promote the onset and progression of DKD (11).

In the microenvironment of DKD, renal cells undergo significant lipid metabolism reprogramming to cope with the metabolic stress induced by hyperglycemia, insulin resistance, and chronic inflammation. First, lipid influx increases, particularly with the upregulation of lipid transporters such as Cluster of differentiation 36 (CD36) and fatty acid transport protein (FATP) (12), leading to excessive uptake and accumulation of free fatty acids. Second, abnormal lipid synthesis is enhanced, with increased activity of key enzymes such as fatty acid synthase (FASN) and acetyl-CoA carboxylase (ACC) (13), further exacerbating lipid accumulation. At the same time, fatty acid oxidation is impaired, with reduced AMPK activity, resulting in the failure to efficiently clear excess lipids and causing lipotoxicity. Moreover, inflammation and hypoxia activate signaling pathways like Nuclear Factor-kappa B (NF-κB) (14), further driving lipid metabolism abnormalities and creating a vicious cycle. These metabolic changes collectively promote the progression of DKD and exacerbate kidney damage.

In recent years, there has been an increasing focus on lipid metabolism reprogramming in the microenvironment of DKD, particularly with the application of high-throughput omics technologies, which have revealed the complex network of metabolic products and pathways involved in DKD.

However, there are still some limitations in current research. For instance, although several signaling pathways and metabolic products associated with lipid metabolism reprogramming have been identified, the precise pathological mechanisms remain unclear. Recent reviews have comprehensively summarized lipid metabolism in kidney disease (15, 16). This review provides a novel synthesis of emerging evidence on microenvironmental crosstalk within the diabetic kidney, elucidating how hypoxia and chronic inflammation cooperatively reprogram lipid metabolism to drive lipotoxicity, and systematically examining the regulatory interplay between lipid peroxidation and programmed cell death. By analyzing and synthesizing the current findings, this review hopes to provide new insights and directions for future basic research and clinical treatment of DKD.

## 2 Landscape and mechanisms of lipid metabolic reprogramming

In DKD, various stages of lipid metabolism, including lipid influx, synthesis, storage, cholesterol efflux, and lipid catabolism, undergo significant alterations, leading to lipid metabolism reprogramming and exacerbating renal injury. First, insulin resistance increases the release of free fatty acids (FFAs) and the uptake of lipids by kidney cells, particularly through the upregulation of fatty acid transport proteins such as CD36, which results in the accumulation of FFAs in the kidneys. At the same time, the fatty acid synthesis pathway is aberrantly activated, with upregulation of key enzymes such as FASN and ACC, leading to excessive lipid synthesis. Excess lipids are stored in lipid droplets within kidney cells, and the accumulation of lipid droplets is closely associated with dysfunction of tubular epithelial cells and podocytes. Additionally, cholesterol efflux mechanisms are impaired, with reduced function of ATP-binding cassette transporters (ABCA1, ABCG1), resulting in excessive intracellular cholesterol accumulation, which further exacerbates renal inflammation. More critically, the β-oxidation of fatty acids in the kidneys is significantly reduced, and mitochondrial dysfunction leads to the accumulation of unoxidized lipids and their metabolic byproducts (such as diacylglycerol and ceramide), disrupting cellular homeostasis, driving lipotoxicity (17), and triggering oxidative stress, fibrosis, and kidney injury. Overall, lipid metabolism reprogramming in DKD exhibits abnormalities at multiple steps, providing a foundation for disease progression and potential targets for future therapeutic strategies.

### 2.1 Lipid influx

Lipid influx refers to the process by which cells acquire lipid molecules from the external environment through various mechanisms. These lipid molecules include fatty acids, cholesterol, and triglycerides, which primarily enter the cell through lipid transport proteins such as CD36 and FATP. Lipid influx plays a crucial role in maintaining cell membrane structure, energy supply, and signal transduction. In DKD, studies have shown that renal cells exhibit significantly increased lipid influx, primarily due to the upregulation of lipid transport proteins.

CD36, as one of the key lipid transport proteins, is significantly upregulated in DKD, leading to increased fatty acid uptake. The mechanism underlying CD36 upregulation may be associated with increased oxidative stress in the hyperglycemic environment.CD36 is the primary system for free fatty acid (FFA) uptake in the kidney, with high expression in proximal and distal epithelial cells, podocytes, and mesangial cells (18). Under hyperglycemic conditions, CD36 has also been shown to promote chronic inflammation, oxidative stress, and fibrosis in proximal tubular cells. Inhibition of CD36 overexpression in human glomerular mesangial cells and diabetic rats can reduce FFA uptake and improve oxidative stress and fibrosis (19). CD36 may represent a promising therapeutic target for DKD. However, lipid toxicity may not solely be attributed to the increased lipid influx, as lipid overload induced by CD36 overexpression does not cause spontaneous renal fibrosis in mice (20). Therefore, further investigation is needed to explore the complex effects of changes in CD36 levels or function during DKD progression.

Fatty acid uptake is mediated by FATPs, and increased fatty acid uptake in proximal tubular epithelial cells is associated with the pathogenesis of DKD (21). The most abundant FATP in the kidney is FATP2, which is primarily expressed at the apical membrane of proximal tubules (22). Mice with a FATP2 gene knockout exhibit normalized glomerular filtration rate, reduced albuminuria, improved renal pathology, and prolonged lifespan. Further studies are required to elucidate the role of FATP2 in DKD (23). Kidney injury molecule-1 (KIM-1), a member of the immunoglobulin superfamily, is predominantly expressed in the proximal tubular epithelial cells. KIM-1 expression is low in normal kidney tissue but increases rapidly on the apical surface of dedifferentiated proximal tubular cells upon kidney injury. KIM-1 mediates the proximal tubular uptake of palmitate-bound albumin, leading to interstitial inflammation, fibrosis, and secondary tubular enhancement of glomerulosclerosis. The small molecule inhibitor TW-37 can inhibit KIM-1, improving injury, suggesting that KIM-1 could be a potential therapeutic target for DKD (24).

**Figure 1:**
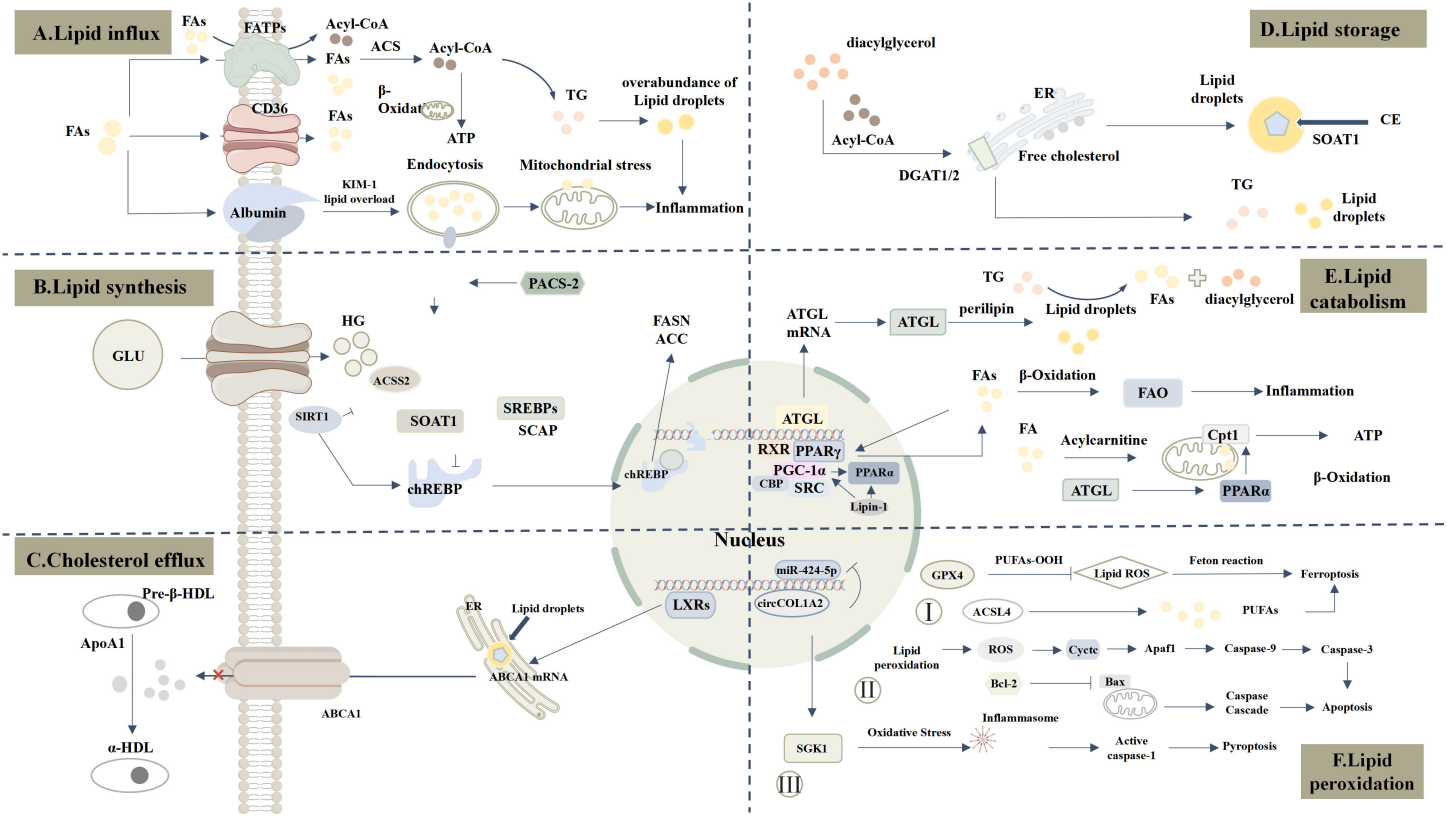
Comprehensive Landscape of Lipid Metabolism Dysregulation in DKD. **(A)** Lipid influx: CD36 and FATPs are significantly upregulated, increasing FAs uptake. KIM-1 mediates palmitate-albumin uptake, further enhancing lipid influx. **(B)** Lipid synthesis: Under hyperglycemia (HG), ChREBP is activated and translocates to the nucleus, promoting FASN and ACC expression and increasing FAs and TG synthesis. Upregulated ACSS2 suppresses SIRT1, amplifying ChREBP activity. PACS-2 regulates SOAT1/SREBPs axis, enhancing lipid synthesis. ACSS2 catalyzes the conversion of short-chain fatty acids into Acetyl-CoA (Ac-CoA), which promotes the formation of the SREBF1/SCAP complex and drives lipid synthesis. **(C)** Cholesterol efflux: ABCA1 binds ApoA1 to mediate HDL transport. In DKD, LXR regulates ABCA1, influencing cholesterol efflux. **(D)** Lipid storage: DGAT1/2 catalyzes the conversion of Acyl-CoA to TG, while SOAT1 esterifies free cholesterol into cholesterol esters stored in lipid droplets. **(E)** lipid catabolism: ATGL downregulation and aberrant Perilipin expression lead to lipid peroxidation product accumulation. Suppression of PPARα and PGC-1α impairs FAO. CPT1 expression and activity are markedly reduced, limiting mitochondrial fatty acid transport and oxidation. **(F)** Lipid peroxidation: 1) Oxidative stress induces lipid peroxidation and inflammasome activation, promoting pyroptosis via SGK1-enhanced oxidative stress. 2) Lipid peroxidation-derived ROS damages mitochondria, releasing Cytochrome c and activating apoptosis pathways. 3) Reduced GPX4 expression promotes lipid peroxides, while ACSL4 activation catalyzes PUFAs oxidation, driving ferroptosis.

Excessive lipid influx leads to the overaccumulation of lipids within renal cells, forming lipid droplets. This lipid accumulation not only directly affects cellular function but also induces inflammation and oxidative stress, further damaging kidney tissue and contributing to cellular dysfunction, inflammation, and fibrosis (**Figure 1A**).

### 2.2 Lipid synthesis and storage

Lipid synthesis primarily refers to the process by which glucose and other substrates are converted into fatty acids and triglycerides within cells through a series of enzymatic reactions. FASN and ACC are key enzymes in the lipid synthesis pathway, and their activity and expression play an important role in regulating lipid synthesis. In the context of hyperglycemia, lipid synthesis pathways in the kidneys undergo significant changes. Studies have shown that in DKD, the expression and activity of FASN and ACC are markedly increased, leading to enhanced endogenous lipid synthesis. This change may be associated with hyperglycemia-induced insulin resistance and oxidative stress.

ACSS2, identified as a critical driver of kidney fibrosis, promotes *de novo* lipogenesis (DNL) by converting acetate to acetyl-CoA, which activates the ACSS2/SREBF1/SCAP axis. ​In this axis, acetyl-CoA facilitates the formation of the SREBF1/SCAP complex, enabling SREBF1 proteolytic processing and nuclear translocation to upregulate lipogenic genes (e.g., FASN, ACC). This process depletes NADPH and increasing ROS levels triggering NLRP3-dependent pyroptosis in renal tubular cells. Pharmacological inhibition of FASN, a downstream target of ACSS2, effectively attenuates lipid accumulation and fibrosis in murine models (25). ​Notably, the ACSS2/SREBF1/SCAP axis is amplified in diabetic kidneys, forming a feedforward loop that exacerbates lipid deposition and fibrosis. ACSS2 interacts with Sirtuin 1 (SIRT1) to suppress its expression. Normally, SIRT1 inhibits ChREBP activity through deacetylation, thereby suppressing the expression of lipid synthesis genes (26, 27). However, when SIRT1 expression is inhibited, ChREBP activity increases, promoting the expression of lipid synthesis genes such as FASN and ACC (28), which exacerbates tubulointerstitial inflammation and mitochondrial oxidative stress. Knockout of the ACSS2 gene reduces renal fatty acid accumulation in diabetic mice and alleviates damage to renal tubules and podocytes (29).

The sterol regulatory element-binding protein (SREBP) pathway is tightly regulated by SREBP cleavage-activating protein (SCAP). Under sterol-depleted conditions, SCAP escorts SREBP-1 from the endoplasmic reticulum to the Golgi apparatus, where sequential proteolytic cleavage releases the transcriptionally active N-terminal domain of SREBP-1 (25). Activated SREBP-1 then binds to sterol response elements (SREs) in the promoters of lipogenic genes (*FASN*, *ACC*), driving triglyceride synthesis and deposition (30). In DKD, hyperglycemia and oxidative stress disrupt this regulatory loop. Studies reveal that SREBF1 (the gene encoding SREBP-1) is transcriptionally upregulated in diabetic kidneys, further amplifying SREBP-1 expression and activity (21, 31). Mechanistically, activated SREBP-1 binds to the *TGF-β* promoter, driving fibrotic signaling—a hallmark of DKD progression (32). This feedforward mechanism promotes mesangial expansion, glomerulosclerosis, and proteinuria—hallmarks of DKD progression (25).

Counteracting this, Phosphofurin acidic cluster sorting protein 2 (PACS-2) mitigates lipid-induced damage by modulating the SOAT1/SREBP axis, highlighting its protective role in maintaining renal lipid homeostasis (33). In summary, under DKD conditions, increased renal lipid synthesis leads to excessive intracellular lipid accumulation, resulting in steatosis and kidney dysfunction.

The process of lipid storage refers to the conversion of excess lipids into lipid droplets, which are stored in the cytoplasm. Recent studies suggest that lipid droplet and *de novo* lipogenesis contribute to oxidative stress and fibrosis, emphasizing the need for interventions targeting lipid storage (16).

Lipid droplets consist of a core of neutral lipids (such as triglycerides and cholesterol esters) surrounded by a monolayer of phospholipids. When cells have excess lipids, they are converted into triglycerides and cholesterol esters in the endoplasmic reticulum, forming lipid droplets. The lipid droplets have been observed in DKD kidney cells, particularly in podocytes and proximal tubular cells (34, 35). Numerous clinical studies have shown that high triglyceride levels are a risk factor for the development of DKD (36, 37). Diacylglycerol acyltransferase 1/2 (DGAT1/2) catalyzes the synthesis of triglycerides from diacylglycerol and acyl-CoA. However, the specific role of triglycerides in DKD cells remains unexplored.

SOAT1 in the endoplasmic reticulum converts free cholesterol into cholesterol esters, which are then stored in lipid droplets. The accumulation of cholesterol esters can induce lipotoxicity and promote DKD. In podocytes, SOAT1 inhibitors can reduce cholesterol ester content in human podocytes. In DKD models, SOAT1 deficiency prevents renal lipid accumulation, protects renal function, reduces proteinuria, and maintains kidney tissue structure (38). Therefore, inhibiting SOAT1 to block cholesterol esterification may represent a novel therapeutic strategy for DKD (**Figures 1B, D**).

### 2.3 Cholesterol efflux

Cholesterol homeostasis is essential for maintaining cellular function, and impaired cholesterol efflux is a key factor in renal pathology. ATP-binding cassette transporter subfamily A member 1 (ABCA1) is a membrane-associated transporter that mediates cholesterol efflux via apolipoprotein A-1, leading to the formation of nascent high-density lipoprotein (HDL) and thereby reducing intracellular cholesterol levels. Type 2 diabetes reduces ABCA1 expression, and ABCA1 deficiency is a susceptibility factor for DKD. Deficiency of ABCA1 in renal cells promotes lipid accumulation, especially cardiolipin in mitochondria, leading to podocyte injury and worsening DKD (39, 40). Pharmacological induction of ABCA1 (e.g., with ABCA1 inducer A30) can improve these conditions. Additionally, 5-arylnicotinamides and cyclodextrin significantly ameliorate renal pathology by upregulating ABCA1-mediated cholesterol efflux. Thus, ABCA1 inducers may offer effective treatment for DKD and other kidney diseases (41, 42).

The transcription of ABCA1 is directly regulated by liver X receptors (LXRs), nuclear receptors that act as cellular “cholesterol sensors” by being activated by natural products of cholesterol metabolism to regulate gene transcription. LXRs are highly expressed in the kidney (43), and LXR agonists can significantly induce ABCA1 mRNA, promoting cholesterol efflux and conversion into lipoproteins (44). In diabetic mouse models, LXR agonists reduce proteinuria, renal inflammation, and cholesterol content in glomeruli by increasing ABCA1 expression, thus playing a crucial role in reducing lipid accumulation within the glomeruli. However, first-generation LXR agonists (T0901317, GW3965) have adverse effects on plasma and hepatic triglyceride levels, limiting their clinical use (45). Fortunately, N-dimethylhydroxycholic amide (DMHCA) is a new-generation gene-selective LXR agonist that does not induce hypertriglyceridemia. Using gene-selective LXR agonists that do not cause hypertriglyceridemia may represent a novel approach to slowing DKD progression (**Figure 1C**).

### 2.4 Lipid catabolism

Fatty acid oxidation is a vital energy source for renal cells, and reduced lipid catabolism capacity leads to lipid accumulation within these cells, further causing metabolic disruption and renal dysfunction. Carnitine O-palmitoyltransferase 1 (CPT1) is the rate-limiting enzyme in fatty acid oxidation, converting fatty acids into acylcarnitine for mitochondrial β-oxidation to produce energy. However, studies tracking type 2 diabetes patients have shown a significant reduction in fatty acid oxidation (FAO), which may be associated with early DKD progression (46). Inhibition of CPT1 exacerbates palmitate-induced podocyte death, whereas CPT1 activators increase FAO and have been shown to help slow kidney disease progression (17). Notably, recent studies on tubule-specific CPT1A knockout models have provided novel insights into this field. Although CPTT1A deficiency reduced mitochondrial fatty acid oxidation capacity by 30%, the kidney maintained energy homeostasis through upregulation of peroxisomal β-oxidation genes (e.g., ACOX1, EHHADH) and activation of the glycolysis-PDK4 axis. This metabolic plasticity explains the absence of expected renal injury severity in clinical observations of CPT1A-deficient patients, suggesting the existence of multi-layered metabolic compensatory networks in the kidney (47). Adipose triglyceride lipase (ATGL) is the rate-limiting enzyme for triglyceride breakdown, essential for lipid catabolism. In ATGL knockout mice, lipid droplets accumulate in podocytes and tubular cells, impairing the renal filtration barrier and reabsorption function. Inhibition of ATGL reduces renal expression of peroxisome proliferator-activated receptor α (PPARα), indicating suppressed lipid metabolism, which leads to lipid deposition, apoptosis in proximal tubules, and ultimately renal fibrosis and dysfunction (48).

Lipin-1 plays a crucial role in maintaining lipid metabolic balance and kidney health, and its dysregulation may be a significant factor in the progression of DKD. As a transcriptional coactivator, Lipin-1 regulates fatty acid oxidation (FAO) through interactions with PPARα (peroxisome proliferator-activated receptor α) and PPARγ coactivator 1α (PGC-1α). During DKD progression, Lipin-1 expression in the kidney initially increases and then decreases. In the absence of Lipin-1, PGC-1α and PPARα-mediated FAO signaling pathways are suppressed, leading to reduced fatty acid utilization and oxidation, lipid accumulation in tubular epithelial cells, and ultimately exacerbating fibrosis and causing renal dysfunction (**Figure 1E**) (49).

### 2.5 Abnormal cell death related to lipid peroxidation

Lipid peroxidation is the oxidative reaction of lipid molecules under the influence of free radicals, producing cytotoxic lipid peroxides. These lipid peroxides not only disrupt the structure and function of cell membranes but also induce apoptosis and necrosis. In DKD, elevated oxidative stress in a hyperglycemic environment increases the production of free radicals (such as superoxide anions and hydroxyl radicals), leading to enhanced lipid peroxidation. Studies have shown that levels of lipid peroxides, such as malondialdehyde (MDA), are significantly elevated in DKD patients. Lipid peroxides exhibit strong cytotoxicity, damaging the structure and function of renal cell membranes and inducing apoptosis and necrosis. Additionally, lipid peroxides activate inflammatory and fibrotic pathways, accelerating kidney disease progression. Thus, lipid peroxidation is not only a key factor in the pathology of DKD but also an important mediator of renal injury.

#### 2.5.1 Ferroptosis

Ferroptosis is a form of programmed cell death characterized by the accumulation of iron-dependent lipid peroxides (50). Glutathione peroxidase 4 (GPX4) is a major regulator of ferroptosis; as an antioxidant defense enzyme, it reduces ferroptosis. In kidney biopsy samples from diabetic patients, GPX4 expression is significantly reduced (51). In animal models of DKD, increasing GPX4 expression through various drugs markedly reduces the accumulation of renal lipid peroxides (52, 53), thereby protecting renal cells. Reducing oxidative damage by increasing GPX4 expression may inhibit ferroptosis, an area of active research. The ferroptosis inhibitor Ferrostatin-1 alleviates TGF-β1-induced ferroptosis in DKD. In diabetic mice, mRNA and protein expression of Cystine/Glutamate Antiporter Light Chain Subunit (xCT) and GPX4 are reduced, glutathione levels decrease, and lipid peroxidation increases. Glutathione, a key intracellular antioxidant, decreases with oxidative stress, leading to ferroptosis. Treatment with Ferrostatin-1 alleviates these changes, suggesting that inhibiting ferroptosis may mitigate diabetes-induced kidney damage. Inhibiting ferroptosis may offer a new approach to treating DKD. The effects of Fer-1 demonstrate the potential of this strategy, and future therapies based on ferroptosis inhibition may help slow or prevent the progression of DKD (51).

Acyl-CoA synthetase long-chain family member 4 (ACSL4) has recently been identified as a crucial component in executing ferroptosis. Activated ACSL4 catalyzes the biosynthesis of lipids containing polyunsaturated fatty acids and promotes the accumulation of lipid peroxidation products, leading to ferroptosis (54). Among insulin sensitizers, rosiglitazone is a strong ACSL4 inhibitor and can target ACSL4 to reduce tissue death in ferroptosis mouse models (55). In DKD mouse models, ACSL4 is primarily expressed in renal tubules. The ACSL4 inhibitor Rosi can improve kidney function by reducing lipid peroxidation and ferroptosis through decreased MDA levels and increased GPX4 mRNA expression (56).

#### 2.5.2 Apoptosis

Lipid peroxidation generates reactive oxygen species (ROS), causing mitochondrial damage, leading to the loss of mitochondrial membrane potential and the release of cytochrome c, thereby activating apoptotic signaling pathways (57). Lipid peroxidation and nitric oxide (NO) are significantly increased in diabetic kidneys, while glutathione, superoxide dismutase, and catalase levels are reduced. In the renal tissue of diabetic rats, Bax and caspase-3 levels are significantly elevated, whereas Bcl-2 is markedly reduced. Statins can reduce lipid peroxidation and nitric oxide, improving the expression of antioxidants and apoptotic markers (58). Chlorogenic acid (CGA), a naturally occurring phenolic compound and one of the most common active components in coffee, directly scavenges free radicals that lead to lipid peroxidation through its strong antioxidant mechanism, significantly inhibiting lipid peroxidation and reducing apoptosis (59).

#### 2.5.3 Pyroptosis

Pyroptosis is a form of programmed cell death dependent on the activation of inflammasomes, such as NOD-like Receptor Pyrin Domain-Containing Protein 3 (NLRP3). While lipid peroxidation is not a direct cause of pyroptosis, it can induce pyroptosis by enhancing oxidative stress, which activates the NLRP3 inflammasome. Serum and Glucocorticoid-Regulated Kinase 1 (SGK1) promotes lipid peroxidation and inflammatory responses by increasing oxidative stress, thereby exacerbating pyroptosis. Circular RNA Collagen Type I Alpha 2 Chain (circCOL1A2) is highly expressed in the plasma of DKD patients and in high-glucose-induced renal tubular epithelial cells *in vitro*. circCOL1A2 promotes oxidative stress and pyroptosis by inhibiting miR-424-5p expression and increasing SGK1 levels. Knockdown of circCOL1A2 restores miR-424-5p expression and suppresses SGK1, thereby reducing oxidative stress and pyroptosis in DKD (60) (**Figure 1F**).

### 2.6 Key signaling pathways affecting lipid metabolism

The expression of various key enzymes in lipid metabolism can be regulated by multiple signaling pathways, including AMP-activated protein kinase (AMPK), mammalian target of rapamycin (mTOR), and peroxisome proliferator-activated receptors (PPARs), which are further modulated by upstream pathways such as Phosphoinositide 3-Kinase/Protein Kinase B (PI3K/AKT) and SIRT1 (61).

#### 2.6.1 AMPK and mTOR signaling pathways

The regulation of anabolic and catabolic processes in cells is coordinated by two primary nutrient sensors—mTOR and AMPK pathways. These signaling pathways have opposite but complementary roles in lipid metabolism and play an essential role in maintaining renal cell homeostasis.

mTOR regulates lipid synthesis by activating the mTORC1. In the high-glucose environment of DKD, mTORC1 is activated, promoting the biosynthesis of fatty acids and cholesterol (62, 63). mTORC1 stimulates fatty acid synthesis by upregulating ACC and FASN and enhances cholesterol synthesis by activating SREBP1. Additionally, mTORC1 activation inhibits fatty acid oxidation by blocking CPT1, leading to more lipid storage within cells for later use (64). However, excessive mTOR activation can result in lipid accumulation and oxidative stress, increasing the risk of kidney damage.

AMPK is activated during energy scarcity, prompting cells to generate ATP through fatty acid oxidation to prioritize energy supply (65). AMPK reduces malonyl-CoA levels by inhibiting ACC, thereby relieving inhibition on CPT1 and enhancing fatty acid oxidation. It also decreases fatty acid and cholesterol synthesis by inhibiting ACC and 3-hydroxy-3-methylglutaryl coenzyme A (HMG-CoA) reductase. Activation of AMPK effectively suppresses metabolic lipid accumulation and helps slow kidney disease progression (66). AMPK can directly inhibit mTORC1 activity by phosphorylating its Raptor subunit, thus supporting catabolic processes, while mTORC1 activation can suppress AMPK activity. The balance between mTOR and AMPK is crucial for maintaining the function of tubular epithelial cells and podocytes (67). In DKD, excessive activation of mTORC1 and reduced AMPK activity lead to lipid metabolism abnormalities and cellular damage (68).

#### 2.6.2 PPAR signaling pathway

PPARs are a family of nuclear receptor transcription factors that include three isoforms: PPARα, PPARβ/δ, and PPARγ. These isoforms are primarily expressed in different tissues, where they perform distinct functions (69).

PPARα is highly expressed in proximal tubules and podocytes, and its activation increases fatty acid metabolism in renal proximal tubular epithelial cells, reducing lipid accumulation and thereby alleviating lipotoxicity in diabetic conditions (70, 71). The PPARα agonist fenofibrate effectively reduces glomerular injury in DKD. Additionally, PPARα regulates the expression of genes related to fatty acid transport and oxidation, such as CPT-1, which helps clear fatty acid accumulation in the kidneys of DKD patients (72).

In DKD, PPARγ expression is associated with renal lipid accumulation, inflammation, and fibrosis. Studies show that PPARγ agonists (such as thiazolidinediones) can improve insulin sensitivity in tubular cells, reduce inflammatory responses, and alleviate lipotoxic damage in tubular epithelial cells by regulating lipid metabolism (73). Activation of PPARβ/δ also promotes fatty acid utilization, but its role in DKD is not well-studied, requiring further investigation to clarify its effects and potential therapeutic applications.

## 3 Cellular lipid metabolism reprogramming in the DKD microenvironment

### 3.1 Immune cells

In the pathogenesis of DKD, macrophage lipid metabolism undergoes significant changes and plays a critical role in disease progression. Macrophages lie at the intersection of lipid metabolism and inflammation, contributing to the remodeling of the DKD microenvironment. Pro-inflammatory M1 macrophages rely on aerobic glycolysis and fatty acid synthesis to promote the production of pro-inflammatory cytokines, further aggravating kidney injury. In contrast, anti-inflammatory M2 macrophages utilize fatty acid oxidation and oxidative phosphorylation to aid in inflammation resolution and tissue repair (74). In the high-glucose microenvironment of DKD, macrophages are more prone to M1 polarization, activating the p38 Mitogen-Activated Protein Kinase (MAPK) pathway, leading to cellular injury and apoptosis. Promoting anti-inflammatory M2 macrophage polarization effectively reduces kidney damage (75).

As lipid sensors, LXRs play a crucial role in macrophage function and the progression of DKD by regulating the expression of genes involved in lipid metabolism and inflammation (76). Activation of LXRs promotes cholesterol efflux from macrophages by upregulating cholesterol efflux genes, such as ABCA1 and ABCG1, reducing intracellular cholesterol accumulation, lipid droplet formation, and foam cell development. Additionally, LXRs regulate genes like SREBP-1c to enhance fatty acid synthesis and breakdown, optimizing intracellular lipid balance. LXRs also inhibit NF-κB activity, reducing pro-inflammatory cytokines such as interleukin-1beta (IL-1β), Tumor Necrosis Factor-alpha (TNF-α), and Interleukin-6 (IL-6), which in turn decreases inflammation and macrophage infiltration in kidney tissue (77). By suppressing ROS production induced by glycosylated or acetylated Low-Density Lipoprotein (LDL), overexpression or activation of LXRs significantly mitigates oxidative stress (78). In hyperlipidemic-hyperglycemic nephropathy models, macrophage-specific expression of LXRα markedly improves pathological changes, reducing inflammatory cell infiltration and fibrosis in the kidneys, thereby alleviating DKD-related renal damage (78).

Triggering Receptor Expressed on Myeloid Cells 2 (TREM2) -high macrophages are a type of lipid-associated macrophage initially identified in immune cells within adipose tissue using single-cell sequencing, and their presence has also been confirmed in kidney biopsy samples from DKD patients, particularly in glomerular and tubulointerstitial regions. Studies indicate that these macrophages increase significantly in response to disrupted lipid homeostasis, especially in diabetic mouse models and high-fat diet-induced diabetic mice (79). TREM2 was initially recognized as a regulator of inflammation and immune response, but recent findings suggest that TREM2 signaling modulates macrophage lipid handling and metabolic capacity (80, 81). In metabolic diseases, TREM2 expression is elevated, promoting the expression of proteins associated with lipid influx, such as LDL receptor (LDLr), CD36, and Lectin-like Oxidized Low-Density Lipoprotein Receptor-1 (LOX-1) (82). Further research is needed to clarify the specific function of TREM2-high macrophages in DKD and their role in lipid metabolism dysregulation.

Macrophages mediate glucolipotoxicity through the myeloid-related protein 8 (MRP8) and Toll-like receptor 4 (TLR4) signaling pathway. Studies have shown that in diabetic mice, mRNA levels of MRP8 and TLR4 are elevated in glomeruli, leading to increased expression of inflammatory and fibrotic factors, thereby exacerbating lipid metabolism disorders and inflammation, ultimately resulting in renal injury and fibrosis. In Tlr4 knockout mice, the effects of a high-fat diet on DKD are abolished (83, 84), suggesting this pathway as a potential target for intervening in DKD progression.

Furthermore, free fatty acids induce tubular epithelial cells to transform into cells with macrophage-like functions, thereby amplifying the local inflammatory response. Under high-fat conditions, tubular epithelial cells acquire macrophage-like characteristics by expressing markers such as CD68, a process mediated by fatty acids (85).

**Figure 2:**
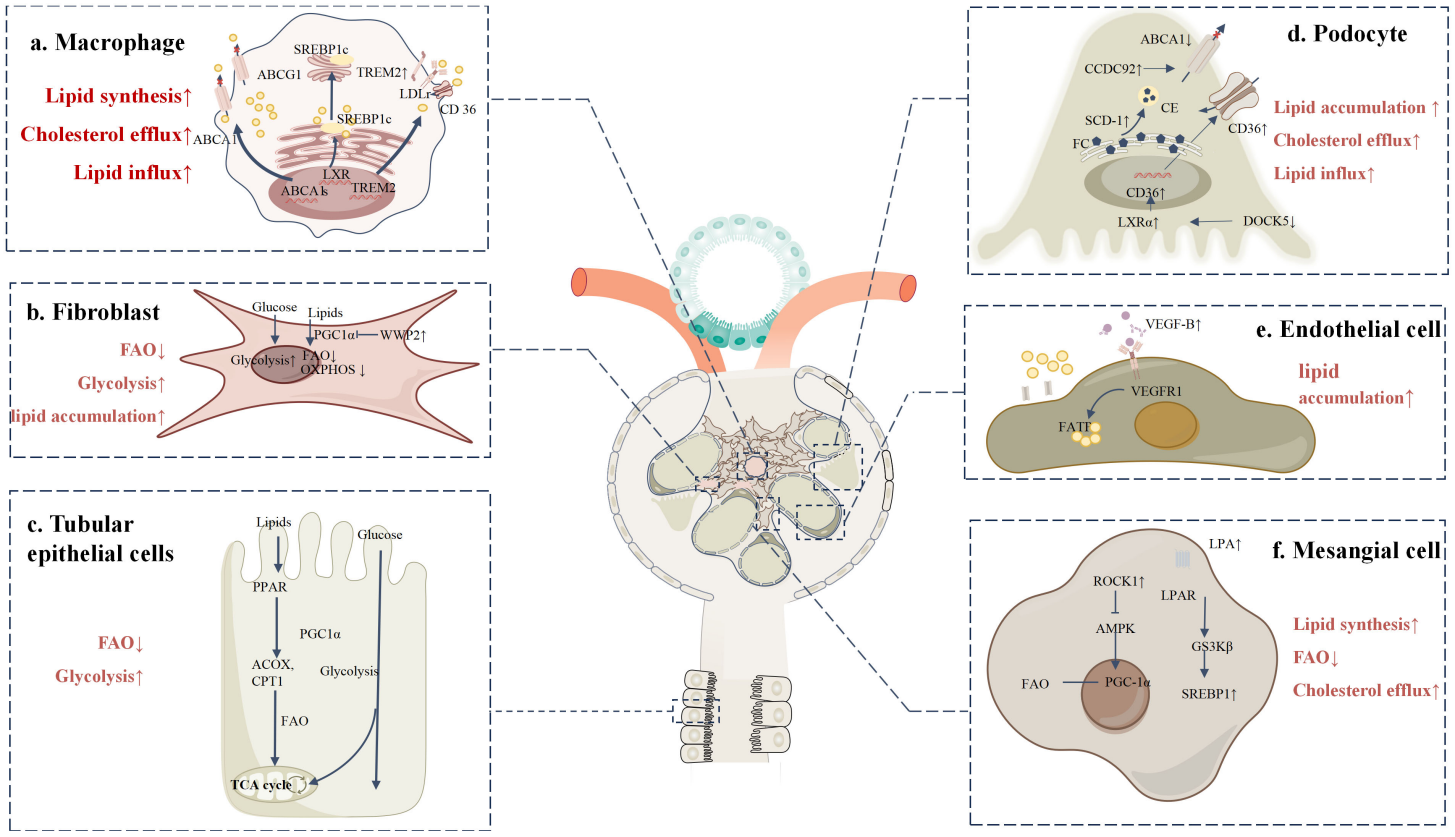
Lipid metabolic reprogramming in various cells within the DKD microenvironment **(a)** TREM2-high macrophages promote the uptake of cholesterol and LDL by upregulating lipid uptake-related proteins, such as LDL receptor, CD36, and LOX-1. Meanwhile, cholesterol efflux pathways are impaired, with reduced expression of ABCA1 and ABCG1, leading to decreased cholesterol clearance and the formation of foam cells. Additionally, LXR activation enhances the expression of genes related to fatty acid synthesis and storage via SREBP. **(b)** Upon activation, fibroblasts exhibit significantly increased WWP2 expression under DKD conditions. WWP2 suppresses mitochondrial bioenergetic regulator PGC1α, inhibiting FAO and OXPHOS, while glycolysis pathways are markedly enhanced. **(c)** In DKD, the expression of key metabolic enzymes such as CPT1, ACOX, and PPARα is downregulated in TECs, preventing efficient mitochondrial fatty acid oxidation. This suppression of FAO leads to lipid accumulation, and metabolic reprogramming shifts TECs toward glycolysis and other alternative energy-generating pathways. **(d)** In podocytes, increased SCD1 activity converts free cholesterol (FC) into cholesterol esters (CE), promoting lipid droplet formation and inducing lipotoxicity. Upregulation of CCDC92 inhibits ABCA1-mediated cholesterol efflux, while decreased Dock5 expression enhances LXRα activity, increasing CD36-mediated fatty acid uptake. **(e)** VEGF-B is overexpressed in DKD and binds to its receptor VEGFR-1, significantly upregulating FATPs, which enhances fatty acid uptake and accumulation. **(f)** In mesangial cells, ROCK1 activation suppresses the phosphorylation of key FAO regulators PGC-1α and AMPK, leading to reduced fatty acid utilization and impaired FAO. Additionally, elevated serum LPA binds to LPAR on the cell surface, activating downstream GSK3β signaling and promoting SREBP1 nuclear translocation, thereby enhancing lipid synthesis.

In the DKD microenvironment, in addition to macrophage accumulation, immune cells such as neutrophils, T cells, and B cells are also significantly enriched. However, few studies have focused specifically on the immune cell level (9). With advancements in single-cell and spatial omics technologies, an increasing number of studies are examining immune cells in DKD, though more detailed research on lipid metabolism in this context is still needed (**Figure 2a**).

### 3.2 Intrinsic cells

In the pathological progression of DKD, renal intrinsic cells exhibit significant alterations in fatty acid metabolism and lipid homeostasis under high-glucose and high-fat conditions, including increased lipid synthesis, inhibited lipid transport and breakdown, and lipid accumulation. These lipid metabolism disorders ultimately lead to lipotoxicity, cellular dysfunction, and further deterioration of kidney function.

Tubular epithelial cells (TECs) are the primary sites of energy metabolism in the kidney, where lipid metabolic reprogramming is especially pronounced. Under healthy conditions, TECs rely mainly on FAO for energy. However, in DKD, FAO is significantly suppressed due to reduced expression of key metabolic enzymes like CPT1, preventing efficient fatty acid transport to mitochondria for oxidation, resulting in lipid accumulation. With FAO restricted, TECs undergo metabolic reprogramming toward glycolysis and the pentose phosphate pathway (PPP) as alternative energy sources. Nevertheless, this adaptive shift is insufficient to compensate for the loss of FAO, leaving cells vulnerable to oxidative stress and contributing to the progression of DKD (10).

Different types of renal cells exhibit varying sensitivity to lipid accumulation, with podocytes, as key components of the glomerular filtration barrier, being particularly susceptible (86). In podocytes, increased activity of key fatty acid metabolism enzymes, such as stearoyl-CoA desaturase 1 (SCD1) and FASN, upregulates lipid synthesis, leading to lipid accumulation (38). Upregulation of CCDC92 induces lipotoxicity in podocytes by inhibiting ABCA1-mediated lipid efflux (87). Additionally, reduced expression of Dock5 upregulates LXRα in an m6-A-dependent manner, enhancing CD36-mediated fatty acid uptake in podocytes (88). The increased expression of lipid transport proteins like CD36 further exacerbates the intake of exogenous fatty acids and abnormal intracellular lipid accumulation. These metabolic abnormalities lead to podocyte structural damage, such as foot process effacement and cellular hypertrophy, ultimately compromising the integrity of the glomerular filtration barrier.

In mesangial cells, activation of rho-associated, coiled-coil-containing protein kinase 1 (ROCK1) inhibits phosphorylation of key metabolic factors in FAO, such as PGC-1α and AMPK, leading to decreased fatty acid utilization (89). Elevated serum lysophosphatidic acid (LPA) in DKD binds to LPA receptors on mesangial cells (90), activating glycogen synthase kinase 3 beta (GSK3β) and promoting nuclear translocation of SREBP1. This activation further enhances lipid synthesis by downregulating smurf2, thereby inhibiting ubiquitin-mediated degradation of chREBP (91). Additionally, LXR-mediated expression of cholesterol efflux transporters ABCA1 and ABCG1 is significantly reduced.

Upregulation of vascular endothelial growth factor beta (VEGF-β) promotes the expression of FATPs, particularly FATP4, thereby increasing fatty acid uptake and accumulation in glomerular endothelial cells (92).

In DKD, fibroblasts undergo a phenotypic transition from a quiescent to an activated state, driving the fibrosis process. Resident renal fibroblasts are the primary source of these cells, with additional sources including epithelial cells, endothelial cells, bone marrow-derived precursor cells, and pericytes (93). Fibroblasts are the main contributors to renal fibrosis. Recent studies using spatial omics and single-cell sequencing have analyzed gene expression in fibrotic regions, identifying various cell types in the fibrotic microenvironment, including fibroblasts, myofibroblasts, immune cells, endothelial cells, and damaged tubular cells (94). WWP2 (an E3 ubiquitin-protein ligase) is significantly upregulated in DKD, promoting fibroblast activation and fibrosis. WWP2 limits FAO and mitochondrial oxidative phosphorylation (OXPHOS) by inhibiting PGC1α expression, reducing fatty acid utilization in fibroblasts. This shifts their metabolism toward glycolysis and lipid accumulation, exacerbating lipid accumulation and fibrosis in the renal interstitium. Targeting WWP2 or activating PGC1α may help restore lipid metabolic balance in fibroblasts and slow fibrosis progression in DKD (**Figure 2**) (95).

## 4 Microenvironmental factors affecting lipid metabolic reprogramming

During the onset and progression of DKD, changes in the renal microenvironment play a crucial role in lipid metabolic reprogramming. Inflammatory responses and hypoxia are primary factors, working through various molecular mechanisms to disrupt lipid metabolism. Below is a detailed analysis of these key microenvironmental factors and their roles in lipid metabolic reprogramming.

### 4.1 Hypoxia

Hypoxia is a critical factor in the pathological mechanisms of DKD. In DKD, metabolic changes in renal cells (96), including increased glucose and sodium reabsorption mediated by Sodium-Glucose Cotransporter 2 (SGLT2) in proximal tubules, lead to significantly higher oxygen consumption. At the same time, factors such as hyperglycemia and mitochondrial dysfunction reduce cellular oxygen utilization efficiency, further increasing cellular oxygen demand (97). Capillary damage in the glomerulus, caused by hyperfiltration and hyperperfusion, results in reduced renal blood flow, which fails to meet the additional oxygen demand, leading to localized tissue hypoxia (94, 98).

Studies have shown that under hyperglycemic and hypoxic conditions, cellular glycolytic pathways undergo significant changes. For example, in HK cells cultured in high-glucose and hypoxic environments, glycolytic products such as pyruvate, lactate, glyceraldehyde-3-phosphate, and glucose are significantly increased, while phospholipid, cholesterol ester, and fatty acid metabolism are reduced, with a notable increase in ceramide levels (99). Noninvasive chemical shift-selective (CSS) imaging and blood oxygen level-dependent (BOLD) magnetic resonance (MR) imaging have revealed a relationship between lipid content and renal oxygenation in diabetic kidneys of db/db mice. Lipid accumulation in diabetic kidneys impairs renal tissue oxygenation, making it more susceptible to renal hypoxia (100).

In a hypoxic environment, the limited oxygen supply prevents full utilization of electrons in the electron transport chain, inhibiting OXPHOS (101). To compensate for the energy deficit, cells increase the rate of anaerobic glycolysis. The primary metabolite of anaerobic glycolysis is lactate, whose accumulation alters intracellular pH, thereby inhibiting fatty acid synthesis and breakdown. Many lipid-metabolizing enzymes are highly sensitive to pH changes, so an acidic environment significantly disrupts fatty acid metabolism (102).

Furthermore, anaerobic glycolysis requires nicotinamide adenine dinucleotide (NAD+) as a coenzyme. Under hypoxic conditions, mitochondrial dysfunction prevents the oxidation of NADH to NAD+ via the electron transport chain, reducing the supply of NAD+ and thereby limiting fatty acid oxidation (103). To meet energy demands, cells must consume more glucose, directing it preferentially toward glycolysis rather than fatty acid synthesis. This shift in metabolic priority inhibits normal lipid metabolism (104). In DKD, mitochondrial dysfunction and an imbalance in mitochondrial fission and fusion dynamics lead to increased mitochondrial fragmentation and reduced mitophagy, resulting in decreased energy production (105). This ultimately causes intracellular fatty acid accumulation, exacerbating lipotoxicity.

In addition to the metabolic effects caused by high glucose, endothelial cells gradually lose their endothelial characteristics and acquire fibroblast-like properties through the process of endothelial-to-mesenchymal transition (EndMT). This process leads to impaired integrity of renal blood vessels, causing vascular leakage and capillary thinning, which results in insufficient oxygen supply to kidney tissue and further exacerbates the hypoxic environment (106–108).

**Figure 3:**
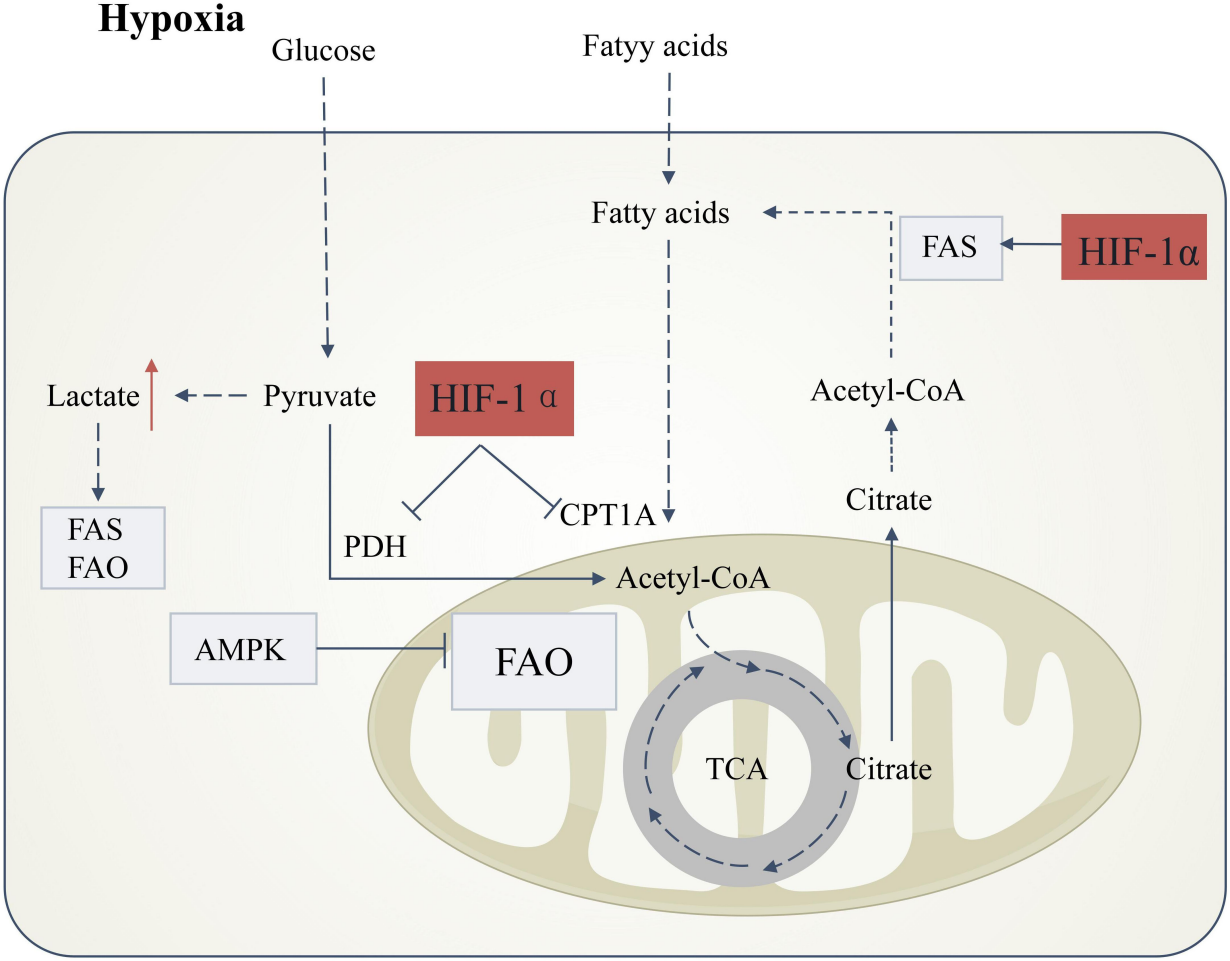
Hypoxia-induced lipid metabolic reprogramming in DKD. Under hypoxic conditions, stabilization of hypoxia-inducible factor-1α (HIF-1α) orchestrates metabolic shifts that drive renal injury. HIF-1α activation suppresses pyruvate dehydrogenase (PDH) activity, blocking pyruvate entry into the tricarboxylic acid (TCA) cycle and redirecting glucose flux toward anaerobic glycolysis. This metabolic rewiring elevates lactate production, inducing intracellular acidification that concurrently disrupts fatty acid synthesis (FAS) and fatty acid oxidation (FAO), thereby promoting lipid accumulation.HIF-1α further exacerbates lipid dysregulation by dual mechanisms: (1) Direct inhibition of carnitine palmitoyltransferase 1A (CPT1A), impairing mitochondrial fatty acid transport and FAO; and (2) Transcriptional activation of lipogenic pathways. These effects are amplified by hypoxia-mediated suppression of AMP-activated protein kinase (AMPK), a master regulator of FAO. Concomitantly, citrate derived from truncated TCA cycling is diverted to fuel FAS, creating a feed-forward loop for lipid deposition. Boxes denote directional changes in molecular activity or metabolite levels: light gray (downregulation) and red (upregulation).

Hypoxia and oxidative stress inhibit AMPK function, which is a key regulator of fatty acid oxidation. The suppression of AMPK activity leads to decreased fatty acid oxidation, thereby exacerbating lipotoxicity (109). Hypoxia-inducible factor (HIFs) are the primary regulators of oxygen homeostasis. Under hypoxic conditions, the HIF-1α pathway is activated, inhibiting pyruvate dehydrogenase, preventing pyruvate from entering the citric acid cycle and oxidative phosphorylation, and promoting glycolysis. This inhibition of oxidative phosphorylation reduces ROS production, thus alleviating hypoxia-induced oxidative stress (110, 111). However, in the long term, this may exacerbate lipotoxicity, as HIF can promote the expression of fatty acid synthase, increasing fatty acid synthesis, while inhibiting the activity of the fatty acid oxidation enzyme CPT1A, reducing fatty acid transport into the mitochondria and lowering fatty acid oxidation efficiency (106, 112–114), thereby increasing lipid deposition (115). Recent studies have also shown that hyperglycemic and hyperlipidemic environments lead to increased expression of HIF-1α and Heme Oxygenase-1 (HO-1) in renal tubular cells, which degrade heme to generate iron, carbon monoxide, and biliverdin, increasing intracellular iron content and promoting lipid peroxidation and ferroptosis (**Figure 3**) (116, 117).

### 4.2 Inflammation

Although DKD is generally classified as a non-inflammatory kidney disease, the inflammatory microenvironment plays a significant role in the progression of DKD (118). In the context of DKD, the main sources of pro-inflammatory mediators that lead to kidney damage are infiltrating macrophages and resident renal cells (119). Renal intrinsic cells, such as podocytes and endothelial cells, also participate in the innate immune response (120, 121). DAMPs activate pattern recognition receptors (PRRs), which are expressed in immune cells (macrophages, dendritic cells (DC), and neutrophils) and non-immune cells (podocytes, epithelial cells, endothelial cells, and mesangial cells) in the kidney (122). Resident and circulating immune cells interact with local renal cell populations, and the release of pro-inflammatory mediators drives the inflammatory response (119, 123). Lipid overload can trigger an inflammatory response in renal cells through multiple pathways (124, 125), modulating a pro-fibrotic phenotype and ultimately causing kidney damage, a process known as lipotoxicity. In fact, immune-inflammatory responses can also induce alterations in lipid metabolism.

#### 4.2.1 Infiltration of inflammatory cells

In DKD, inflammatory cells such as macrophages and T cells infiltrate kidney tissue in large numbers. These cells secrete various inflammatory mediators and chemokines, directly affecting renal cell function. For example, pro-inflammatory factors such as TNF-α and IL-1β released by M1 macrophages can activate signaling pathways like NF-κB and JAK/STAT, regulating the expression of lipid metabolism-related genes and leading to lipid metabolism dysregulation.

Macrophage accumulation in the kidneys is associated with the progression of type 2 DKD in db/db mice. The accumulation and activation of macrophages in diabetic db/db kidneys are linked to chronic hyperglycemia, glomerular immune complex deposition, and increased production of renal chemokines (126).

High-cholesterol diet-induced hypercholesterolemia exacerbates proteinuria in diabetic rats, accompanied by macrophage infiltration in the glomerulus. Treatment with macrophage colony-stimulating factor (M-CSF) inhibits macrophage infiltration in the glomeruli and urinary protein excretion in hypercholesterolemic diabetic rats. These results suggest that macrophages play a key role in lipid-induced diabetic nephrotoxicity, and M-CSF, as a preventive factor, effectively blocks this process (127).

#### 4.2.2 The role of inflammatory factors

Inflammatory factors such as TNF-α and IL-1β are significantly elevated in DKD (128), and these factors influence lipid metabolism through multiple mechanisms.

TNF-α significantly increases the expression of long-chain acyl-CoA synthetase 1 (ACSL1) in HK2 cells. TNF-α treatment not only increases the levels of short-chain acylcarnitine and long-chain acyl-CoA, but also promotes the conversion of fatty acids into complex lipids (129). TNF-α reduces ABCA1-mediated cholesterol efflux and SOAT1-mediated cholesterol esterification, leading to free cholesterol-dependent apoptosis in podocytes. This process is exacerbated in ABCA1 knockout mice and can be partially prevented by cyclodextrin, indicating that local TNF, independent of serum TNF, TNFR1, or TNFR2 levels, is sufficient to induce lipid metabolic reprogramming in DKD (130).

Inhibitors of the interleukin-1 (IL-1) signaling pathway, such as recombinant human IL-1 receptor antagonist (anakinra) or anti-IL-1β antibodies, can alleviate podocyte death induced by Fetuin-A and palmitic acid. This study suggests that IL-1 signaling induces lipid metabolism dysregulation in DKD (131). Under normal conditions, cells tightly regulate the uptake of low-density lipoprotein (LDL) through the LDL receptor (LDLr) pathway to prevent excess cholesterol accumulation. However, the presence of IL-1β disrupts this feedback mechanism, significantly increasing LDLr expression and function. IL-1β upregulates the mRNA levels of nuclear SREBP-1 and SREBP cleavage-activating protein (SCAP), enhancing the transport of the SCAP-SREBP complex from the endoplasmic reticulum to the Golgi apparatus, thereby activating the transcription and expression of LDLr. This process has been confirmed in both podocytes and mesangial cells and remains active even under high LDL conditions, significantly increasing LDL uptake and exacerbating lipid accumulation in these cells (132–136).

IL-1β not only promotes lipid influx but also inhibits cholesterol efflux mechanisms. IL-1β downregulates the expression of PPARs and LXR, indirectly reducing ABCA1 levels, which leads to decreased cholesterol efflux from podocytes and mesangial cells, further exacerbating intracellular cholesterol accumulation and lipotoxicity (132, 135). Additionally, IL-1β activates the CXCL16 signaling pathway, promoting lipid accumulation in the renal tubulointerstitial region and worsening interstitial damage. As a scavenger receptor, CXC motif chemokine ligand 16 (CXCL16) facilitates the uptake of oxidized low-density lipoprotein (oxLDL). IL-1β upregulates the expression of CXCL16, A Disintegrin And Metalloproteinase 10 (ADAM10), and CXC Chemokine Receptor 6 (CXCR6), significantly increasing lipid accumulation, reactive oxygen species (ROS) production, and extracellular matrix (ECM) secretion, thereby exacerbating tubulointerstitial injury. These findings suggest that the CXCL16 pathway plays a critical role in IL-1β-mediated lipid metabolic reprogramming and cellular damage (137).

IL-1β also exacerbates lipid metabolism dysregulation in DKD through its synergistic interaction with other pro-inflammatory signaling pathways. For example, blocking VEGF-B and IL-17A signaling significantly alleviates kidney damage and improves renal function, not only reducing lipid deposition (particularly neutral lipids) but also decreasing local inflammation (138).

In addition, the complement system, as a crucial component of the innate immune system, rapidly generates a large number of protein fragments upon activation. These fragments act as effective mediators of inflammation, vascular activity, and metabolic responses, thereby promoting the progression of DKD (139, 140). Complement C5a is a potent pro-inflammatory mediator. In DKD, excessive activation of complement C5a through its receptor C5aR1 leads to abnormalities in renal lipid metabolism pathways and changes in mitochondrial bioenergetics (141). Elevated C5a levels promote alterations in lipid metabolism in diabetic kidneys, increasing the expression of DGAT-1 and SREBP-1, which results in lipid accumulation (142). Targeted inhibition of the C5a/C5aR axis can alleviate inflammation, extracellular matrix deposition, and macrophage infiltration, thereby improving renal function (143).

#### 4.2.3 The role of inflammasomes in lipid metabolism

In DKD, the activation of inflammasomes such as NLRP3 serves as a bridge between inflammatory factors and lipid metabolism dysregulation. Activation of the NLRP3 inflammasome also induces the upregulation of lipid synthesis-related genes, such as SREBP1 and SREBP2, while inhibiting the expression of lipid efflux proteins like ABCA1 (135). This change in gene expression patterns leads to the abnormal accumulation of cholesterol and triglycerides in the glomerulus, exacerbating renal lipotoxicity. Inflammasomes sense lipotoxic signals (such as free fatty acids and oxidative stress), activating caspase-1 and promoting the maturation and release of IL-1β. This induces the activation of ROS (reactive oxygen species) and NF-κB p65, forming a positive feedback loop that further drives lipid accumulation (135).

**Figure 4:**
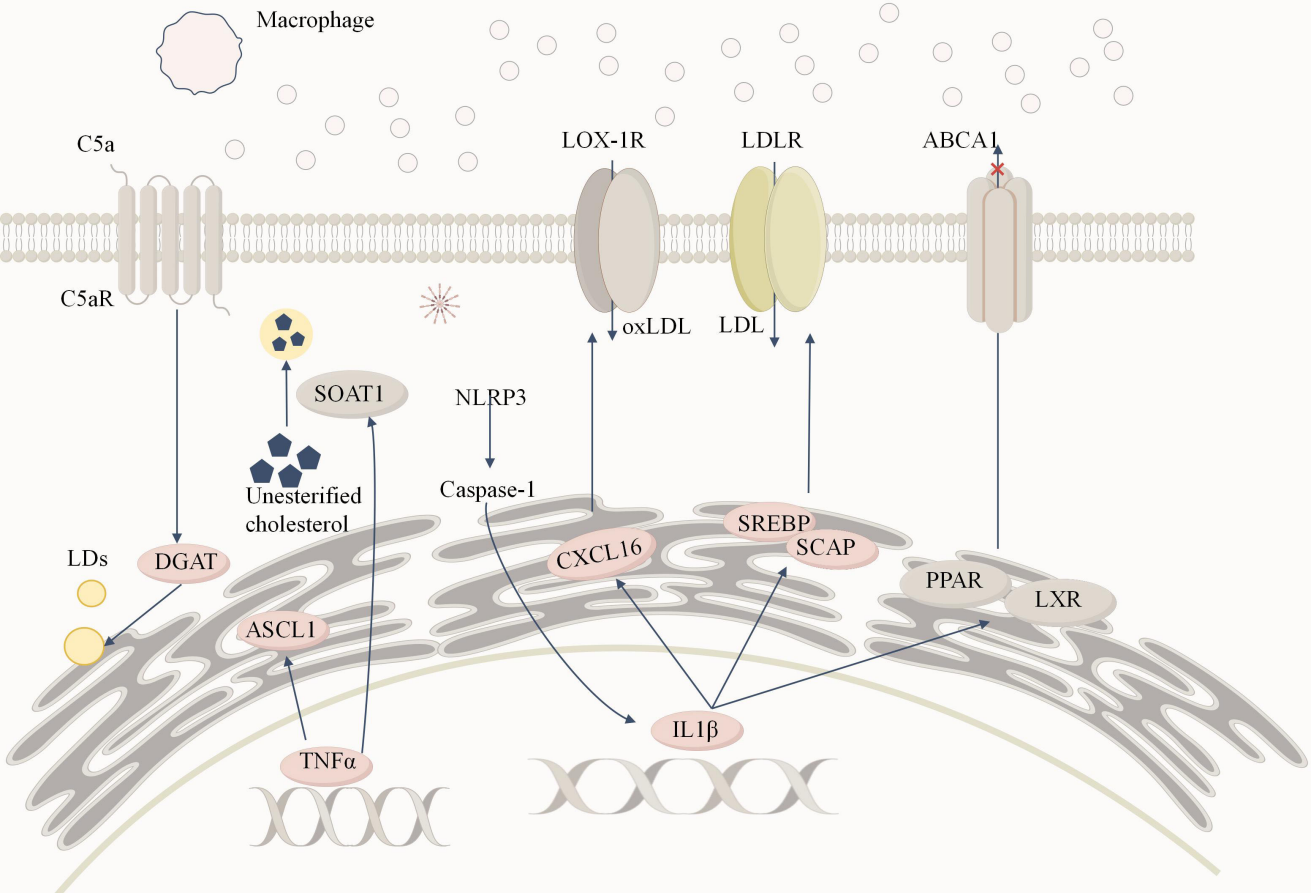
Inflammation-induced lipid metabolism reprogramming in DKD. Macrophages infiltrate renal tissue and release large amounts of TNFα and IL-1β, creating a persistent inflammatory microenvironment. TNFα significantly increases the expression of ACSL1. Additionally, TNFα regulates sterol SOAT1to convert free cholesterol into cholesterol esters, storing them in lipid droplets and contributing to lipid accumulation.IL-1β activates the expression of SREBP and SCAP, enhancing LDLR-mediated uptake of LDL, leading to cholesterol accumulation in podocytes and mesangial cells. Moreover, IL-1β inhibits the activity of nuclear receptors such as PPAR and LXR, reducing ABCA1 (ATP-binding cassette transporter A1)-mediated cholesterol efflux, further exacerbating intracellular cholesterol accumulation. Additionally, IL-1β promotes oxidized LDL (oxLDL) uptake via the CXCL16 pathway, triggering reactive oxygen species (ROS) generation and enhancing extracellular matrix deposition, ultimately causing tubulointerstitial damage. The binding of C5a to its receptor C5aR activates the complement system, which in turn stimulates diacylglycerol acyltransferase 1 (DGAT1), promoting triglyceride formation and increasing intracellular lipid droplet (LD) accumulation. The activation of the NLRP3 inflammasome serves as a critical link between inflammatory cytokines and lipid metabolism disorders. Its activation promotes caspase-1 activation and IL-1β release, forming a positive feedback loop that amplifies inflammatory responses and lipid accumulation, further exacerbating renal lipotoxicity and fibrosis.

Inflammation and lipid overload act synergistically, exacerbating the pathological progression of DKD and leading to further decline in renal function and irreversible damage. The interaction between the inflammatory microenvironment and lipid overload creates a vicious cycle, causing continuous deterioration of DKD (**Figure 4**).

## 5 Targeting lipid metabolism in DKD treatment strategies

Based on a comprehensive understanding of lipid metabolism in DKD, targeting lipid metabolism has been proposed as a promising therapeutic strategy aimed at improving treatment outcomes by regulating lipid metabolism in both renal parenchymal cells and immune cells. However, due to the high plasticity of the lipid metabolic system, when one metabolic pathway is inhibited, the compensatory effects through other pathways may diminish the efficacy of drug treatments. For example, current pro-oxidative therapies represented by CPT1 activators exhibit anti-fibrotic effects in animal models. However, the peroxisomal compensatory mechanisms revealed by CPT1A knockout studies suggest that complete inhibition of mitochondrial FAO may unexpectedly activate alternative lipotoxic pathways. This explains why CPT1 activators only partially ameliorate renal injury in preclinical studies (47). Therefore, current research is focused on upstream targets of lipid metabolism, with the goal of providing a more comprehensive intervention by affecting multiple metabolic processes. Several drugs with nephroprotective effects have demonstrated potential in reducing lipid metabolic reprogramming. For example, Fenofibrate activates the AMPK signaling pathway, inhibits chREBP and SREBP, reduces lipid synthesis, and prevents lipid accumulation and related inflammatory responses in the kidneys (144). Similarly, Dapagliflozin inhibits HIF1α, suppresses lipid catabolism, and regulates lipid metabolism, alleviating lipotoxicity and oxidative stress (104, 116). These drugs have shown significant renoprotective effects in both experimental studies and clinical trials, highlighting the potential value of targeting lipid metabolism in DKD treatment. Although no drugs specifically targeting lipid metabolism for DKD have yet been successfully translated into clinical applications, these findings provide a strong theoretical foundation and practical evidence for the development of effective therapeutic strategies in the future. The table below presents current studies on drugs targeting lipid metabolic reprogramming and their corresponding effects (**Table 1**).

**Table 1:** Summary of drugs targeting lipid metabolic reprogramming and their mechanisms in DKD models.

Target	Treatment	Model	Mechanism	Ref
Lipid influx				
CD36	Fisetin	HFD/STZ LDLR-/- mice,HK-2 cells	PPARγ/CD36 Pathway Inhibition	(145)
	Colquhounia root tablet	STZ SD rats,HK-2 cells	CD36 Inhibition and AMPK Activation	(146)
	Apocynin	HFD/STZ mice	Inhibition of CD36-Dependent Wnt/β-Catenin Activation	(147)
	SS31	db/db mice,HK-2 cells	Suppression of oxidative stress and CD36	(148)
	Astragaloside IV	HFD/STZ SD rats,HK-2 cells	CD36-mediated NLRP3 inflammasome activation	(128)
	CD36 antagonist,SSO	HK-2 cells	CD36 Inhibition Preventing HG-Induced EMT	(149)
	Ginsenoside Rg1	NRK-52E cell	CD36/TRPC6/NFAT2 Inhibition	(150)
FATP2	Astragaloside IV	HFD/STZ SD rats	Inhibition of Fatty Acid Transport, Restoration of Mitochondrial Function	(151)
KIM-1	TW37	KIM-1Δmucin mice,LLC-PK1 kidney epithelial cells	PA-Albumin Uptake inhibition, Renal Inflammation Alleviation	(24).
Lipid synthesis				
chREBP	LPAR1/3 Antagonists ki16425	db/db mice,SV40 MES13	ROS/Akt-dependent downregulation of Smurf2	(91)
	Fenofibrate	db/db mice,NMS2 mesangial cells	AMPK-PGC-1α-ERR-1α-FoxO3a signaling activation	(144)
ACSS2	S8588(ACSS2 inhibitor)	ACSS2-KO mice,STZ,mouse podocytes	Reduction of Raptor/mTORC1-Mediated Autophagy Inhibition	(152)
		STZ/ACSS2 knockdown mice,HK-2 cells	Inhibition of ChREBP-mediated fatty acid lipogenesis, mitochondrial oxidative stress, and inflammatory response	(28)
SREBP	Fenofibrate	db/db mice,NMS2 mesangial cells	AMPK-PGC-1α-ERR-1α-FoxO3a signaling activation	(144)
	Liraglutide	HFD/Unilateral Nephrectomy/STZ SD rats	Inhibition of lipid synthesis and promotes lipolysis,Promotion of AMPK Phosphorylation	(153)
	Leptin	HFD/STZ induced SD rats,NRK-52E	Activation AMPK to attenuate ELD	(154)
	LY294002(PI3K/Akt pathway inhibitor)	STZ CD1 mice,HKC	Increased FBXW7 expression and decreased SREBP-1 expression	(155)
	Curcumin	STZ SD rats	AMPK-SREBP pathway, reduction of renal triglyceride accumulation	(156)
		STZ Wistar rats	Reduction of SREBP-1, iNOS, and TGF-β1 Levels	(157)
	Thymol	HFD mice	Downregulation of SREBP, Reduction of Lipid Accumulation	(158)
	LPAR1/3 antagonis ki16425	db/db mice,SV40 MES13	Blockage of GSK3β phosphorylation and SREBP1 activation	(90)
	FXR agonists GW4064	db/db mice,mesangial cell	Inhibition of SREBP-1c and other lipogenic genes	(159)
	Dapagliflozin	HFD mice	Decrease of SREBP-1c mRNA	(160)
	1α,25(OH)2 D3	NRK-52E	Downregulation SREBPs, Inhibition of lipid accumulation	(161)
	Bilirubin	STZ SD rats	TG synthesis inhibition	(162)
Lipid storage				
DGAT-1	2-phenyl-5-trifluoromethyloxazole-4-carboxamide	HFD mice	Inhibition of DGAT-1 to reduce lipid synthesis and improve insulin resistance	(163)
SGLT2	SGLT2i JNJ 39933673	db/db mice	Inhibition of SGLT2 to reduce renal lipid accumulation and inflammation	(164)
	Empagliflozin	db/db mice	Inhibition of AGEs-RAGE to alleviate cholesterol accumulation	(165)
Cholesterol efflux				
ABCA1	Captopril+Spironolactone	STZ SD rats	Targeting the ABCA1 and mir-33 gene expression	(166)
	Exendin-4	apoE-/- mice,human renal glomerular endothelial cells	Increase of ABCA1to alleviating renal lipid accumulation, inflammation, and proteinuria	(167)
	ABCA1 inducer A30	db/db mice	Induction of ABCA1 to Reduce Oxidative Stress, Decrease Albuminuria, and Restore Podocyte Foot Process	(40)
LXRα	Anthocyanins	HK-2 cells	PPAR α-LXR α-ABCA1 activation	(168)
	LXR agonist T0901317	db/db mice	downregulation of FXIII-A expression	(169)
		STZ mice	Promotion of ABCA1 and ABCG1 to enhance cholesterol efflux	(170)
	LXR agonist DMHCA	STZ/HFD mice,Lxrα/β (-/-) mice	Downregulation of glomerular lipids and plasma triacylglycerol	(171)
Lipid catabolism				
HIF-1α	Dapagliflozin	STZ CD-1 mice	HIF-1α-mediated metabolic switch from lipid catabolism to glycolysis	(104)
CPT1a	C75	FA injection male mice	Increases Cpt1,Block fatty acid synthase	(47)
Lipid peroxidation				
AMPK/NRF2	Empagliflozin	STZ/HFD mice,HK-2 cells	Promotion of AMPK/NRF2 activation pathway to improve lipid peroxidation and prevent ferroptosis	(172)
HIF1α/HO1	Dapagliflozin	db/db mice,HK-2 cells	Alleviation of HIF1α/HO1-Mediated Ferroptosis	(116)

## 6 Discussion

Lipid metabolism reprogramming in the microenvironment of DKD plays a central role in the pathological progression. As diabetes progresses, renal cells adapt to the oxidative stress and inflammatory response induced by hyperglycemia, with abnormal lipid metabolism reprogramming playing a key role in this process. Lipid accumulation, particularly lipotoxicity caused by lipid metabolism dysregulation, has been shown to significantly increase in DKD patients. This accumulation not only leads to cellular dysfunction but also exacerbates endoplasmic reticulum stress and ROS generation, further intensifying cellular damage and inflammation.

Lipid metabolic pathways, including FAO and lipid synthesis, are significantly altered in DKD, leading to reduced fatty acid oxidation capacity and increased lipid accumulation. Dysregulated lipid metabolism, through the induction of pro-inflammatory cytokines and oxidative stress, further drives renal fibrosis and functional decline. The polarization of macrophages within the DKD microenvironment exacerbates this process. The regulatory roles of mTOR and AMPK in lipid metabolism reprogramming warrant further investigation. Abnormal activation of the mTOR signaling pathway and reduced AMPK activity together result in lipid metabolic imbalance. Overactivation of mTOR promotes lipid synthesis and storage, while reduced AMPK activity weakens fatty acid oxidation, thereby aggravating lipid accumulation. Modulating these signaling pathways can alleviate renal lipotoxicity and help mitigate the progression of DKD.

In addition, microenvironmental factors such as hypoxia and chronic inflammation influence lipid metabolism by activating pathways like HIF-1α and NF-κB, further driving lipid metabolism reprogramming. These factors exacerbate kidney cell damage and accelerate the pathological progression of DKD by altering gene expression and enzyme activity within metabolic pathways. Research on microenvironmental factors may offer new therapeutic directions for DKD.

Therapeutic strategies targeting lipid metabolism have shown potential in the treatment of DKD. By inhibiting lipid synthesis or promoting fatty acid oxidation, lipid accumulation in the kidneys can be effectively reduced, alleviating lipotoxicity. Future research should focus on targeting key metabolic regulators and combining these approaches with anti-inflammatory agents to optimize treatment strategies for DKD. This integrated therapeutic approach can not only improve lipid metabolism disorders but also reduce the immune-inflammatory responses induced by these disturbances, thus offering a novel direction for DKD treatment.

Diabetes has traditionally been considered a non-inflammatory disease, but recent studies have highlighted the significant role of immune inflammation in DKD. Although immune cells in the kidney are relatively sparse and challenging to study in depth, it is clear that immune inflammation plays a crucial role in the progression of DKD. In recent years, advances in technology, particularly single-cell RNA sequencing (scRNA-seq) and spatial transcriptomics (spRNA-seq), have led to breakthroughs in the study of the kidney immune system. Using spRNA-seq and scRNA-seq technologies, researchers can more accurately define the DKD microenvironment and identify key areas critical to disease progression. ScRNA-seq reveals the roles of different cell types in the pathological process of DKD, while spRNA-seq precisely maps these cells and their gene expression profiles, further clarifying the spatial distribution of disease progression. This high-resolution analysis helps identify potential therapeutic targets and provides a more detailed basis for treatment strategies. By analyzing cell-to-cell communication and interactions, researchers can better understand the role of cell-cell interactions within the DKD microenvironment. For example, studies may reveal specific cell populations interacting within kidney tissue, offering new targeted therapeutic directions. This deeper understanding of the interactions between immune cells and renal parenchymal cells will aid in developing more precise treatment strategies (9).

Overall, lipid metabolism reprogramming plays a complex and profound role in the DKD microenvironment. Further elucidating its mechanisms will provide crucial insights for developing more effective therapeutic strategies. Dual intervention targeting both lipid metabolism and immune inflammation will be an important direction for future DKD treatments.
